# Alteration in DNA-binding affinity of Wilms tumor 1 protein due to *WT1* genetic variants associated with steroid - resistant nephrotic syndrome in children

**DOI:** 10.1038/s41598-022-12760-x

**Published:** 2022-05-24

**Authors:** Martin Bezdicka, Filip Kaufman, Ivana Krizova, Alzbeta Dostalkova, Michaela Rumlova, Tomas Seeman, Karel Vondrak, Filip Fencl, Jakub Zieg, Ondrej Soucek

**Affiliations:** 1 Vera Vavrova Lab/VIAL, Department of Pediatrics, Second Faculty of Medicine, Charles University and Motol University Hospital, V Uvalu 84, 150 06 Prague, Czech Republic; 2 Department of Biotechnology, University of Chemistry and Technology, Prague, Czech Republic; 3 Department of Pediatrics, Second Faculty of Medicine, Charles University and Motol University Hospital, Prague, Czech Republic

**Keywords:** Clinical genetics, Sequencing, Kidney diseases, Proteins, Experimental models of disease, Genetics research, Paediatric research

## Abstract

Approximately one third of children with steroid-resistant nephrotic syndrome (SRNS) carry pathogenic variants in one of the many associated genes. The *WT1* gene coding for the WT1 transcription factor is among the most frequently affected genes. Cases from the Czech national SRNS database were sequenced for exons 8 and 9 of the *WT1* gene. Eight distinct exonic *WT1* variants in nine children were found. Three children presented with isolated SRNS, while the other six manifested with additional features. To analyze the impact of *WT1* genetic variants, wild type and mutant WT1 proteins were prepared and the DNA-binding affinity of these proteins to the target EGR1 sequence was measured by microscale thermophoresis. Three WT1 mutants showed significantly decreased DNA-binding affinity (p.Arg439Pro, p.His450Arg and p.Arg463Ter), another three mutants showed significantly increased binding affinity (p.Gln447Pro, p.Asp469Asn and p.His474Arg), and the two remaining mutants (p.Cys433Tyr and p.Arg467Trp) showed no change of DNA-binding affinity. The protein products of *WT1* pathogenic variants had variable DNA-binding affinity, and no clear correlation with the clinical symptoms of the patients. Further research is needed to clarify the mechanisms of action of the distinct WT1 mutants; this could potentially lead to individualized treatment of a so far unfavourable disease.

## Introduction

Nephrotic syndrome (NS) is a kidney disease caused by increased permeability of the glomerular filtration membrane. Among the most important building blocks of the membrane are podocytes, disturbances of which play a major role in disease pathophysiology^1^. NS is one of the most common glomerulopathies in children, with an incidence estimated to be 1:25,000 children/year^2^. According to the clinical and laboratory response to an initial four-week long steroid treatment, patients are divided into steroid-sensitive or steroid-resistant cases (about 20% of children)^3^. Children with the steroid-resistant NS (SRNS) are at high risk of progression to end-stage renal disease and increased mortality^4^. Therefore, research clarifying the causes of SRNS and potentially allowing for targeted and personalized treatment is needed to improve such children's prognosis and quality of life.

More than 50 genes have been found to be associated with SRNS to date, and about 30% of children with SRNS carry pathogenic variants in one of these genes^5,6^. The *WT1* gene, one of the most frequently mutated^5,6^, encodes a transcription factor that is important for normal urogenital development, proper formation of nephrons and the maintenance of podocyte function^7,8^. The pathogenic variants of *WT1*may be associated with isolated SRNS cases, but also with SRNS cases that additionally present with urogenital malformations and cancer, and a worse prognosis^9^.

To bind the DNA, WT1 uses the zinc-finger domains (ZFs)^10^. The EGR1 consensus sequence was the very first DNA sequence found to be targeted by WT1^11^ and is present in the regulatory regions of many genes directed by the WT1^7,12^. Interestingly, almost all exonic pathogenic variants of the *WT1* gene are located in exons 8 and 9 that encode for ZFs 2 and 3^13^. Therefore, it is likely that structural alterations of these ZFs may affect the binding affinity between the WT1 protein and EGR1 DNA sequence, potentially leading to change in the transcriptional activity of the protein.

In this functional study, we aimed to describe the DNA-binding affinity of wild type and mutant WT1 proteins selected based on the *WT1* exonic variants found in Czech children with SRNS. These WT1 mutants were recombinantly produced, purified, and their EGR1 binding affinity assessed by a novel molecular interaction analysis method, microscale thermophoresis.

## Results

### Clinical, laboratory and genetic characteristics of children with *WT1* pathogenic variants

Important clinical data for the cases with SRNS are shown in Table 1. The majority of cases (78%) manifested the disease within the first year of life (infantile NS), with only two patients manifesting later at preschool age (median: 5.0 months, min: 8 days, max: 4.2 years). The most frequent symptoms at disease onset were limb and/or facial edema (56% of patients), hypertension (44%) and hematuria (44%). Three (33%) patients had SRNS without any other renal or extrarenal disorders. Three patients (33%, two boys and one girl) had features of Denys-Drash syndrome, i.e. presented SRNS with nephroblastoma (Wilms tumor) and, in the two males, disorders of sex development (cryptorchism). One patient presented with SRNS and hypospadia and a cleft scrotum. The remaining two patients were biamnial monochorionic twins and presented with SRNS and stenosis of the pulmonary artery. The progression to end-stage renal disease was observed in all cases with variable age at presentation ranging from birth to 7.8 years (median 8.0 months). Kidney transplantation was performed in three patients (33%), who are the only patients currently still alive (total mortality was 67%). None of the patients presented with oligohydramnion. Molecular genetic analysis revealed eight different heterozygous *WT1* variants in the nine children with SRNS. All of these variants were previously described in clinical case reports, however mostly without performing a proper functional study (see Table 2).

**Table 1:** Clinical characteristics of patients with *WT1* variants.

Patient No	Gender	Age at disease onset	Gestational age at birth (weeks)	Initial Glomerular filtration rate (ml/min/1.73m^2^)	Protein-creatinine ratio (mg/mmol)	Initial serum albumin (g/L)	Hypertension at the disease onset	Hematuria at the disease onset	Edema at the disease onset	Wilms tumor	Extrarenal manifestation	Renal biopsy finding	Treatment (sorted chronologically)	Time to ESRD	Transplantation	Current health status
1	F	8 months	40	43	515	21.9	No	No	Yes	No	No	DMS	Prednison/Cyclophosphamide	1.9 years	Yes	Alive (now 20 years old)
2 twins	F	8 days	32	13	8 750	22.4	No	No	Yes	No	Stenosis of pulmonary artery	DMS	Methylprednisolon/Conservative treatment/CVVHD	8 days	No	Deceased (due to sepsis, multiple organ failure 5 months after the diagnosis)
3 twins	F	3 weeks	32	90	25 000	19.0	No	Yes	Yes	No	Stenosis of pulmonary artery	DMS	Methylprednisolon/Conservative treatment/PD/CVVHD	2 weeks	No	Deceased (due to sepsis, multiple organ failure 11 months after the diagnosis)
4	F	1.6 years	39	7.5	2 121	25.5	Yes	No	No	No	No	FSGS	CVVHD	21 months	No	Deceased (due to sepsis, multiple organ failure 8 months after the diagnosis)
5	F	5 months	39	90	3 211	29.0	No	Yes	No	No	No	Not indicated	Prednison/Conservative treatment/PD	8 months	No	Deceased (due to hypertensive crisis 15 months after the diagnosis)
6	M	5 months	42	90	1 076	28.0	No	No	No	Yes	DDS, cryptorchism	Not indicated	HD	7.8 years	Yes	Alive (now 18 years old)
7	M	4.2 years	32	5	1 374	27.5	Yes	Yes	Yes	Yes	DDS, cryptorchism	Not indicated	CVVHD	0 days	No	Deceased (due to tumor generalization 1 year after the diagnosis)
8	M	1 week	37	9	7 961	26.0	Yes	Yes	No	No	cleft scrotum, hypospadia	Not indicated	Conservative treatment	0 days	No	Deceased (due to the parents choice of conservative management 2 weeks after the diagnosis)
9	F	8 months	40	90	736	24.0	Yes	No	Yes	Yes	DDS	FSGS	Conservative treatment/HD	1.1 years	Yes (2x)	Alive (now 23 years old)

F = female; M = male; DDS = Denys-Drash syndrome; DMS = diffuse mesangial sclerosis; FSGS = focal segmental glomerulosclerosis; CVVHD = continuous veno-venous hemodialysis; HD = hemodialysis, PD = peritoneal dialysis, ESRD = end-stage renal disease.

**Table 2:** Details on *WT1* found variants.

Patient No	Patient No in^6^	Nucleotide change (NM_024426.5)	Amino acid change (NP_077744.4)	*WT1* exon number	Affected zinc finger	ACMG evaluation	dbSNP reference number (rs)	HGMD number	First published according to HGMD	Previous functional study	MST assay prediction	Binding affinity (Kd, mean ± sd; μM)	Binding affinity difference to wild type	p - value
1	2010	c.1298G > A	p.Cys433Tyr	8	2	LP (PM1, PM2, PP2, PP3, PP5)	No rs number	CM930740	^36^	No	No binding affinity change	89.4(7.0)	Insignificant	3.482161e-01
2, 3	2004a, b	c.1316G > C	p.Arg439Pro	8	2	VUS (PM1, PM2, PP2, PP3, PP5, BS2—positive father)	No rs number	CM114500	^48^	No	Reduced binding affinity	319.0 (72.7)	2.9x	1.248711e-03
4	2048	c.1340A > C	p.Gln447Pro	8	2	P (PS2, PM1, PM2, PM5, PP2, PP3)	No rs number	CM188109	^6^	No	Increased binding affinity	24.9(4.2)	4.4x	3.686159e-04
5	–	c.1349A > G	p.His450Arg	8	2	P (PS2, PM1, PM2, PM5, PP2, PP3)	No rs number	CM941408	^49^	No	Reduced binding affinity	878.5(164.2)	7.9x	6.735329e-03
6	2008	c.1387C > T	p.Arg463Ter	9	3	P (PVS1, PM1, PM2, PP2, PP3, PP5)	rs121907909	CM971596	^50^	Predicted to induce a large splicing change^51^	Reduced binding affinity	323.5(13.3)	2.9x	8.565044e-05
7	2025	c.1399C > T	p.Arg467Trp	9	3	LP (PM1, PM2, PP2, PP3, PP5)	rs121907900	CM910411	^9^	Reduced binding affinity^52^	No binding affinity change	94.6(9.8)	Insignificant	8.497590e-02
8	–	c.1405G > A	p.Asp469Asn	9	3	P (PS2, PM1, PM2, PP2, PP3, PP5)	rs28941778	CM910413	^9^	No	Increased binding affinity	12.1(1.7)	9.3x	9.340891e-03
9	1993	c.1421A > G	p.His474Arg	9	3	LP (PM1, PM2, PP2, PP3, PP5)	No rs number	CM061235	^13^	No	Increased binding affinity	48.4(6.0)	2.3x	7.263909e-03

All variants were found in a heterozygous state and all are absent in databases of global minor allele frequencies. The statistical significance of the difference in mean dissociation constants (Kds) was verified by two-sample t-test computed in R software (p – value).

WT1 wild type Kd (MST) = 110.9 μM (mean ± sd = 7.4 μM).

P = pathogenic variant; LP = likely pathogenic variant; VUS = variant of uncertain significance; ACMG = American College of Medical Genetics and Genomics standards; HGMD = Human Gene Mutation Database.

### WT1 protein binding affinity to EGR1 DNA motif

**Figure 1:**
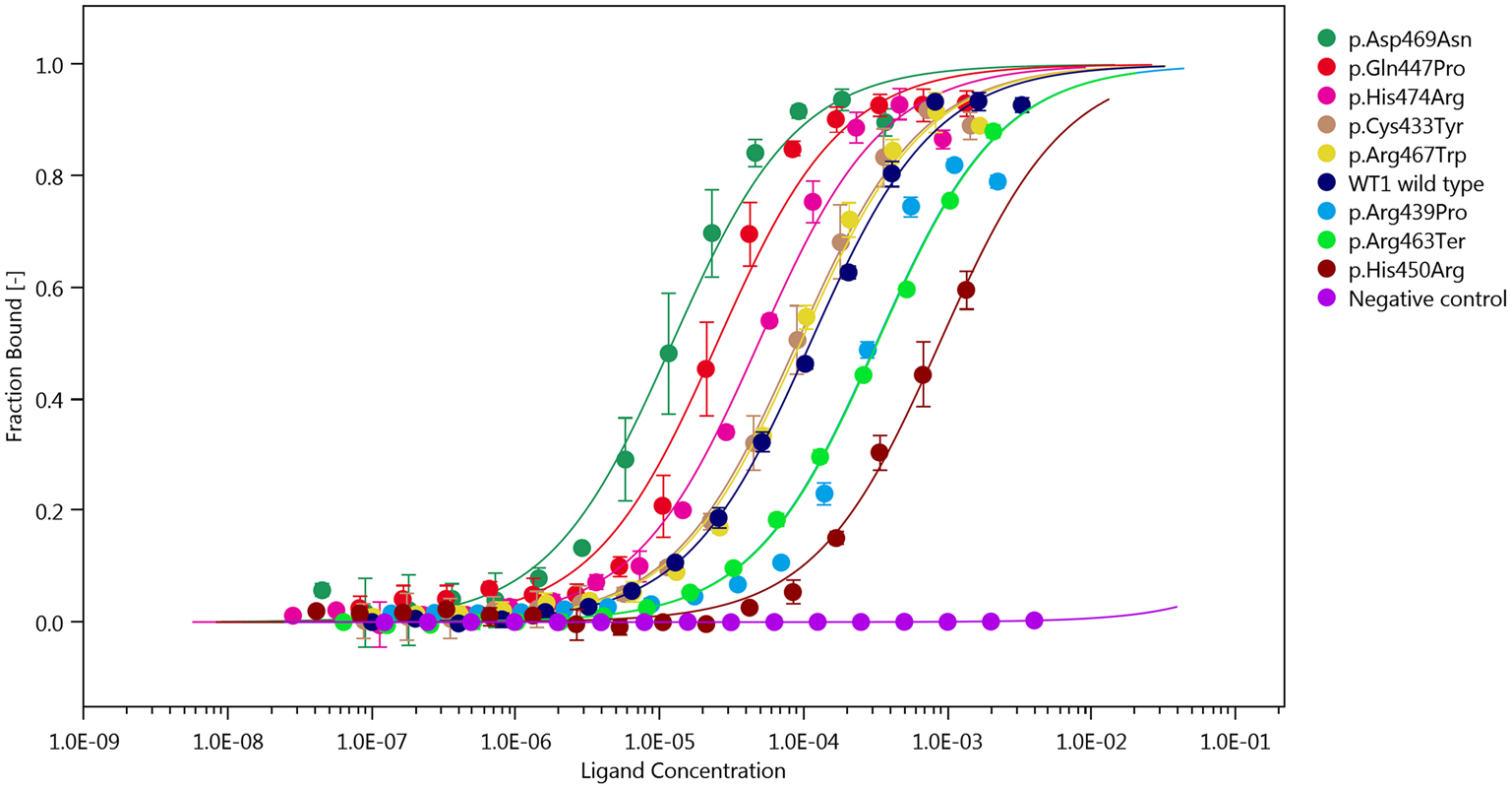
Binding affinity curves of all tested WT1 proteins. The relative amount of WT1 protein bound to the EGR1 DNA motif (fraction bound, y axis) analyzed by microscale thermophoresis. With increasing protein concentration (x axis) the bond becomes saturated (y axis). Shift to the left from the wild type (dark blue) reflects an increased binding affinity, while shift to the right means a decreased affinity. Bovine serum albumin (purple) was used as a negative control. All proteins measured in triplicates.

The affinity curves and dissociation constants (Kds) for each of the WT1 proteins are presented in Fig. 1 and Table 2, respectively. Three WT1 mutants (p.Arg439Pro, p.His450Arg and p.Arg463Ter) showed significantly reduced binding affinity compared to the wild type WT1 protein (all three p < 0.01), whereas three other WT1 mutants (p.Gln447Pro, p.Asp469Asn and p.His474Arg) had increased binding affinity compared to the wild type WT1 protein (all three p < 0.01). In two other WT1 mutants (p.Cys433Tyr and p.Arg467Trp) the analysis did not reveal a statistically significant difference in the binding affinity compared to the wild type WT1 protein. The group of patients studied was too heterogeneous to perform any analyses exploring the association between binding affinity and clinical phenotype. There was no apparent segregation of distinct clinical phenotypes of the cases based on the DNA-binding affinity of WT1 mutants.

**Figure 2:**
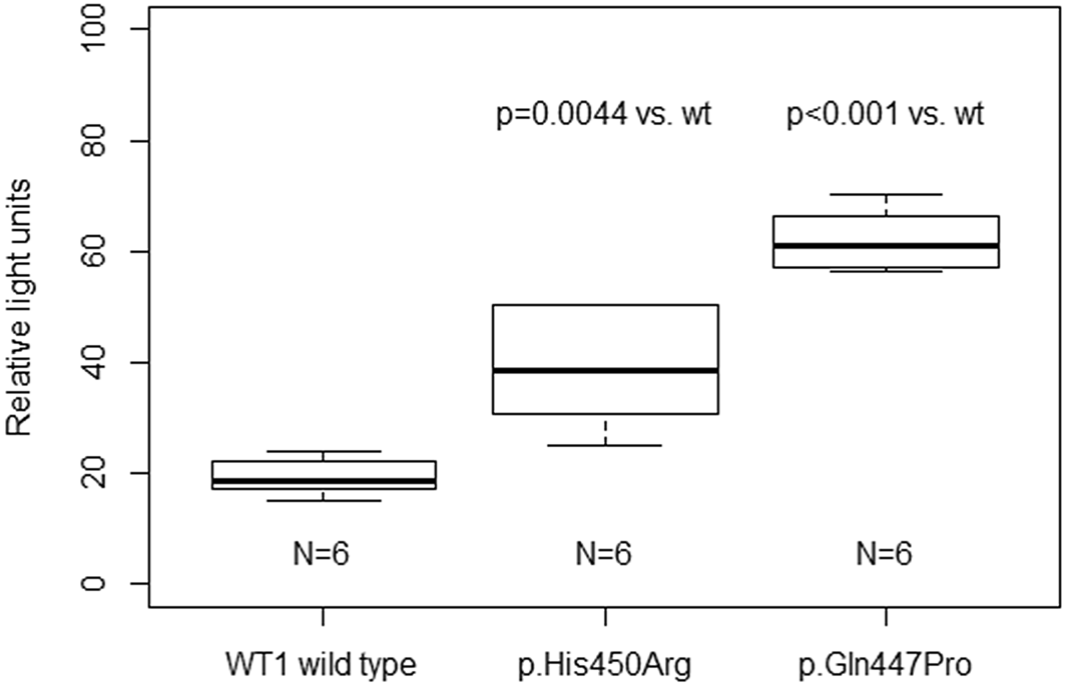
Differential *ACTN1* gene expression assessed by luciferase reporter assay. The effect of WT1 protein variants on *ACTN1* gene expression assessed by luciferase reporter assay. Both WT1 mutant proteins significantly enhanced *ACTN1* expression. The firefly luminescence values were normalized to background (negative control, i.e. vector free HEK293 cells) and to *Renilla* luminescence to adjust for variance in transfection.

To improve our understanding of the link between the DNA-binding affinity of WT1 mutants and the expression of target genes, we performed reporter luciferase assay. Actin gene (*ACTN1*) was identified to be abundantly expressed in kidney according to Human Protein Atlas (https://www.proteinatlas.org/ENSG00000072110-ACTN1/tissue) and also contains WT1-specific binding site in its promoter, very close to the transcription start site^8^, thus posing a feasible target gene. Two representative WT1 mutants were selected (p.Gln447Pro and p.His450Arg, the highest and lowest binding affinity in the MST assay, respectively). The luciferase assay results showed that both WT1 mutant proteins significantly increased *ACTN1* expression compared to WT1 wild type protein (Fig. 2).

## Discussion

This functional in vitro study described the DNA-binding affinity of mutated forms of transcription factor WT1 found in the national cohort of children with SRNS. This might open up a pathway to elucidating the mechanism of action and, consequently, to targeted individualized treatment of the disease, which currently has a poor prognosis and high mortality rate.

### Is *WT1-*pathogenic variant-caused SRNS distinguishable from other monogenic SRNS cases?

While edema, a diagnostic criterion of NS, was missing in 44% (4/9) of the studied patients with exonic *WT1* variants, hypertension, a hugely non-specific symptom, was also detected in 44% (4/9) of these patients. These observations were in agreement with a unique genotype–phenotype study by Lipska et al., where edema was missing in 35% (14/40) and hypertension was noted in 50% (20/40) of patients with SRNS and pathogenic exonic *WT1* variants^14^. Both numbers were much larger in the SRNS cases caused by the *WT1* pathogenic variants compared to the non-*WT1* SRNS cases^14^. Only three patients in the present study (33%, all girls) were classified as “isolated SRNS” cases, i.e. did not show any other renal or extra-renal disorders or symptoms. The frequency (28%) as well as the sex (females only) of these “isolated SRNS” cases was also in agreement with the study by Lipska et al.^14^.

Pathogenic variants in the *WT1* gene were found to be associated with Denys-Drash syndrome, in which SRNS is accompanied by nephroblastoma, disorder of sex development and testicular/ovarian cancer^15^. In the present study, three patients (33%, two boys and one girl) had features of Denys-Drash syndrome. Both boys with Denys-Drash syndrome manifested with SRNS, cryptorchism and nephroblastoma and the girl had SRNS and nephroblastoma without any congenital defects of the genital system. This is in agreement with the Denys-Drash syndrome phenotype in females who usually do not present with disorder of sex development^16^. Cell culture studies showed that WT1 targets the *SRY* gene, which encodes the testis-determining factor on chromosome Y^17^. Mutant WT1 proteins transcribed from the *WT1* gene that contained the pathogenic variants found in Denys-Drash syndrome cases failed to activate the *SRY* promoter in a reporter gene assay, whereas the wild type WT1 protein was able to do so^17^. The *SRY* activation is important for the development of testes and thus determines the male sex^18^; *WT1* variants may thus cause impairment of proper male sex development, which also explains why the disorder of sex development in Denys-Drash syndrome is rather male specific.

Besides the disorder of sex development in males, Denys-Drash syndrome is also characterized by the presence of nephroblastoma (also called Wilms tumor) and testicular/ovarian cancer^15^. These cancer types do not occur in SRNS cases caused by genes other than *WT1*^5^. It was observed that WT1 directly regulates factors DAX1 and SF1, both important in testicular and ovarian development^19,20^. Moreover, transgenic mouse embryos carrying homozygous *WT1* pathogenic variant have shown abrogation of gonadal development^21^. Therefore, impairment of WT1-transcriptional activity may pose a risk factor for the development of testicular and ovarian cancer through the dysregulation of DAX1 and SF1 proteins. Similarly, the loss of WT1 affects Wnt signalling and IGF2 expression, both factors that are important for kidney development, and their dysregulation was found to be present in nephroblastomas^22^. However, approximately 40 different genes have been found to be associated with the development of nephroblastoma^23^ and testicular/ovarian cancer is also not a specific feature of Denys-Drash syndrome. Therefore, although the presence of nephroblastoma and/or gonadal cancer in SRNS cases strongly suggests that *WT1* pathogenic variants are involved in the pathogenesis of the disease, the exact molecular mechanism remains to be elucidated.

Three other children with SRNS also additionally presented with distinct extra-renal features. The monochorionic biamnial twins carrying the p.Arg439Pro variant had a stenosis of pulmonary artery, which is a unique observation, as there has been no such a *WT1*–caused SRNS case published in the literature so far. During heart development, epithelial cells are released from the epicardium into the myocardium and transform into cardiovascular progenitor cells, which can differentiate into coronary wall smooth muscle cells, endothelial cells, perivascular and cardiac interstitial fibroblasts or cardiomyocytes^24,25^. A key activator of the so called epithelial-to-mesenchymal transition process is the Snail factor, which is regulated by the WT1 protein^24^. The Snail factor has been found to be reduced in the *WT1*-knockout immortalized epicardial cells^24^. In addition, another study has found that WT1 together with its corepressor BASP1 inhibited the epithelial cell activator WNT4 in epicardial cells^26^. The epithelial cell activators act against the epithelial-to-mesenchymal transition process and are important for the mesenchymal-to-epithelial transition process (typical for kidney development)^27^. Therefore, decreased WT1 transcription activity present at a specific embryonic stage of heart development may well cause congenital heart defects due to the diminished number of progenitor cardiovascular cells.

The last patient manifested with SRNS and a cleft scrotum and hypospadia. This patient died 3 weeks after birth (two weeks after the diagnosis was confirmed). Some patients with Denys-Drash syndrome develop nephroblastoma months after the manifestation of SRNS^15^. It may thus be speculated that the patient could have developed nephroblastoma later had he survived longer, which would classify him as another case of Denys-Drash syndrome in this case series.

In summary, children with SRNS and a pathogenic variant in the *WT1* gene frequently presented with hypertension and a lack of edemas. A substantial proportion of these patients concurrently manifested disorder of sex development and/or nephroblastoma. These features make this group of patients distinguishable from the other monogenic causes of SRNS.

### The effect of *WT1* variants on the DNA-binding affinity of its protein products

As all of the patients in this study carried *WT1* exonic variants (exons 8 and 9) potentially affecting the structure of the WT1 ZFs 2 and 3, which together with ZF4 have been shown to be responsible for specific DNA sequence recognition^10^, one could expect that the mutations would negatively influence the binding affinity of the protein to its target DNA sequence^13^. Interestingly, three different trends in target DNA binding (reduced, wild type-like and enhanced) were observed for the distinct *WT1* variants.

Decreased DNA-binding affinity of the WT1 protein mutants leads to reduced activity of several genes, i.e. *WNT4*^26^. The WNT4 is the main cell-differentiation factor that drives the mesenchyme-epithelial transition in nephrogenesis^28^. During this process, the metanephric mesenchyme, in close cooperation with the neighboring ureteric bud, induces the formation of renal epithelium and, later, the nephrons^28^. It is known that WT1 expression in metanephric mesenchyme precedes WNT4 expression, and also that the *WNT4* gene carries a specific WT1 DNA binding domain^26^. Moreover, in a cell expression study it has been verified that knockout of *WT1* in mouse embryonic kidney mesenchymal cells is associated with the loss of WNT4 expression^26^. Thus, reduced DNA-binding and the related decrease in the transcriptional activity of WT1 may negatively influence kidney development through abnormal nephron formation. The fetuses of the WT1 null mouse model did not develop kidneys, gonads or spleen, and died at mid-gestation due to defective coronary vasculature^21^. A transgenic adult mouse model with a doxycycline-inducible podocyte-specific knockout of *WT1* has shown progressive albuminuria, FSGS on kidney histological sections and disruption of podocyte structure accompanied by loss of podocalyxin and nephrin, i.e. proteins essential for the glomerular filtration membrane^29,30^. WT1 ortholog knockout in zebrafish embryo has shown pericardial and yolk edema, reduction of kidney size and damage of podocyte foot processes with a poorly developed glomerular filtration membrane^31^. These features were rescued by injection of human wild type WT1 mRNA but not with the *WT1* pathogenic variant^31^. Moreover, HEK293 cells transfected with a *WT1* exonic variant demonstrated downregulation of nephrin and synaptopodin (an important factor for inter-podocyte communication)^31^. Altogether, these studies suggest that the decreased DNA-binding affinity of several of our WT1 mutants may cause the SRNS through the structural and functional impairment of podocytes.

In three patients the WT1 mutants (p.Gln447Pro, p.Asp469Asn and p.His474Arg) showed an increased binding affinity to the EGR1 DNA domain when compared to the wild type WT1 protein. Murine myeloblastic leukemia cells transfected with the *WT1* gene and injected into mice has shown a significant reduction of tumorigenesis compared to none-transfected leukemic cells^32^. WT1 has also induced apoptosis of the primary osteosarcoma cells through activation of the proapoptotic gene *Bak*^33^. In contrast, overexpression of WT1 has been observed in nephroblastoma, breast and colon cancer or acute myeloid leukemia^34,35^. These studies suggest that the WT1 transcription factor may act as both a tumor suppressor and tumor inducer, probably based on concentration and cellular microenvironmental conditions. While under normal conditions WT1 is necessary for proper podocyte differentiation^31^, we can speculate that the same factor may cause podocyte damage through increased transcriptional activity, if the *WT1* sequence variant leads to production of WT1 protein with augmented DNA-binding affinity. Unfortunately, this hypothesis cannot be confirmed, because there have been no studies published focusing on the impact of *WT1* overexpression on kidney development.

The proof-of-concept functional study employing a luciferase assay demonstrated that both WT1 protein mutant with the highest DNA-binding affinity (p.Gln447Pro) as well as the mutant with the lowest affinity (p.His450Arg) enhanced *ACTN1* gene expression, compared to the effect of the WT1 wild type protein. It has been well established that gene transcription in eukaryotic organisms is under combinatorial control of multiple transcription factors and that also conformational changes, not just the DNA-binding itself, significantly influence the assembly and proper function of the general transcription factors and RNA polymerase machinery^36,37^. Therefore, our study shows that alterations in DNA-binding affinity of WT1 protein mutants do not really predict changes in target gene expression and suggest it might be the conformational WT1 protein changes that affect assembly of the transcription machinery complex.

There were two patients with *WT1* variants (p.Cys433Tyr and p.Arg467Trp) who showed no change in the DNA-binding affinity of the resulting WT1 mutants when compared to the affinity of the wild type WT1 protein. Both variants have previously been described as causing syndromic (Denys-Drash syndrome) and also isolated SRNS^9,38^. The WT1 protein has two important isoforms. The WT1 KTS minus isoform is the canonical transcription factor that has much higher binding affinity to the target DNA domain than the WT1 KTS plus isoform^34^. The WT1 KTS plus isoform is formed by alternative RNA splicing, which results in the addition of lysine (K), threonine (T) and serine (S) between ZFs 3 and 4. The KTS insertion changes the rigid ZF conformation, which is thus more flexible and partly prevents ZF4 from its DNA binding^34^. As it has been shown that the WT1 KTS plus isoform is capable of interaction with some RNA binding proteins, and thus can be employed in the splicing machinery or in the regulation of mRNA stability^34,39^, we cannot exclude that the pathogenic effect of both variants has other mechanism than alteration of the DNA binding. However, only the WT1 KTS minus isoforms were produced in this study, which was thus not designed to test the molecular interaction of the different WT1 isoforms. Another possible mechanism may be the interaction between WT1 and its cofactors (i.e., p53, STAT3, BASP1), which modulate WT1 transcription activity^34,40^. If the two *WT1* variants impaired the cofactor-binding site but retained the DNA-binding site of the WT1 mutant, no change would be seen in the binding assay despite the alteration in its transcriptional activity. As the molecular interaction assay was not designed to check for the binding between the distinct WT1 mutants and the WT1 transcriptional cofactors, this possibility can neither be confirmed nor refuted. Although SRNS is known to be a monogenic disease, it may also be that there are risk variants or polymorphisms in the WT1 cofactors that could change the transcriptional activity of the mutant WT1, similarly to the situation in patients with atypical haemolytic uremic syndrome^41^.

In conclusion, this is the first study describing the DNA-binding affinity changes of WT1 mutants found in a national cohort of children with SRNS using a novel molecular interaction analysis method, microscale thermophoresis. Our observations confirm that children with SRNS carrying *WT1* exonic variants quite often present with hypertension and lack edemas, which may help to distinguish them from patients with the other monogenic causes of SRNS. The experimental study found that the distinct WT1 mutants present with variable change in the DNA-binding affinity. Moreover, DNA-binding affinity did not correspond to target gene expression. Multiple factor combinatorial control of gene transcription and conformational changes of WT1 mutants possibly affecting the assembly of transcription machinery complex could explain the lack of clear link between WT1 gene mutation, DNA-binding of its protein product and target gene expression. There was no clear association between the change in the DNA-binding affinity and the clinical phenotype. Although evidence is discussed regarding the possible mechanisms of action of the variable DNA-binding affinity and the multisystemic effects of WT1 mutants, there is a lack of detailed knowledge on how distinct *WT1* variants cause the disease. To enable personalized treatment and improve the unfavourable prognosis of the patients, further investigation is needed to explore the mechanisms of action of the distinct WT1 mutants.

## Methods

### Identification of WT1 gene variants

Within the Czech national database of children with SRNS, a total of nine *WT1* gene positive cases were identified from eight unrelated families (two patients were monochorionic biamnial twins). The study was approved by the institutional Ethics Committee of Motol University Hospital and we confirm that all methods were carried out in accordance with relevant guidelines and regulations. The study subjects and their guardians provided their informed consent in writing. All cases had a negative family history of kidney disease and were born from non-consanguineous marriages. Seven patients were included in our previously published study of the genetic causes of SRNS^6^, where a two-tiered approach was implemented (Sanger sequencing of *NPHS1*, *NPHS2* and *WT1* genes as a first step, and then, when negative, next-generation sequencing of a 48-gene-panel). Since the publication of that paper, two further patients have been identified by routine Sanger sequencing of the four most frequently mutated genes in SRNS cases (i.e. *NPHS1*, *NPHS2*, *WT1* and *NUP93*). The clinical significance of the identified variants was assessed using well-known prediction programs (Mutation Taster, Provean, Polyphen-2, Human Splicing Finder, UMD predictor and CADD scores). The Human Gene Mutation Database^42^ was searched for previous descriptions of the variants, and the NCBI dbSNP for global minor allele frequencies (exclusion of common variants with a frequency of 1% or more in healthy populations found in 1000 Genomes, GnomAD and NHLBI ESP Genomes). Current standards as published by the American College of Medical Genetics and Genomics were followed to evaluate variant pathogenicity. A freely available online software tool that implements these standards was used for the evaluation of the revealed variants^43,44^.

### Preparation of WT1 proteins

All the preparation methods are described in detail in the Supplementary material. The DNA manipulations were carried out using standard subcloning techniques, and plasmids were propagated in *E. coli* DH5α^45,46^. The (–KTS) isoform of the *WT1* gene was obtained by reverse transcription of total RNA isolated from A549 cells using RNeasy mini kit (Qiagene). The *E. coli* BL21 (DE3) CodonPlus RIL strain was transformed by wild type or each mutant *WT1* plasmid by using heat-shock. The isolation of WT1 proteins from the bacterial pellet was achieved by using buffers with increasing concentrations of sodium chloride. The prepared sample was subjected to fast liquid protein chromatography (FPLC, ÄKTA pure protein purification system with Unicorn software) to separate the proteins according their ion charge (HiPrep SP FF 16/10 by Cytiva, strong cation exchange chromatography column). The achieved protein was then separated using high-resolution preparative gel filtration chromatography (HiLoad 26/600 Superdex 200 pg column by Cytiva). The concentration of purified WT1 proteins was calculated and before the MST experiment the samples were concentrated (see Supplementary material for details). The sample buffer was exchanged for the MST binding assay buffer and the final assay concentration was calculated.

### Binding analysis

**Figure 3:**
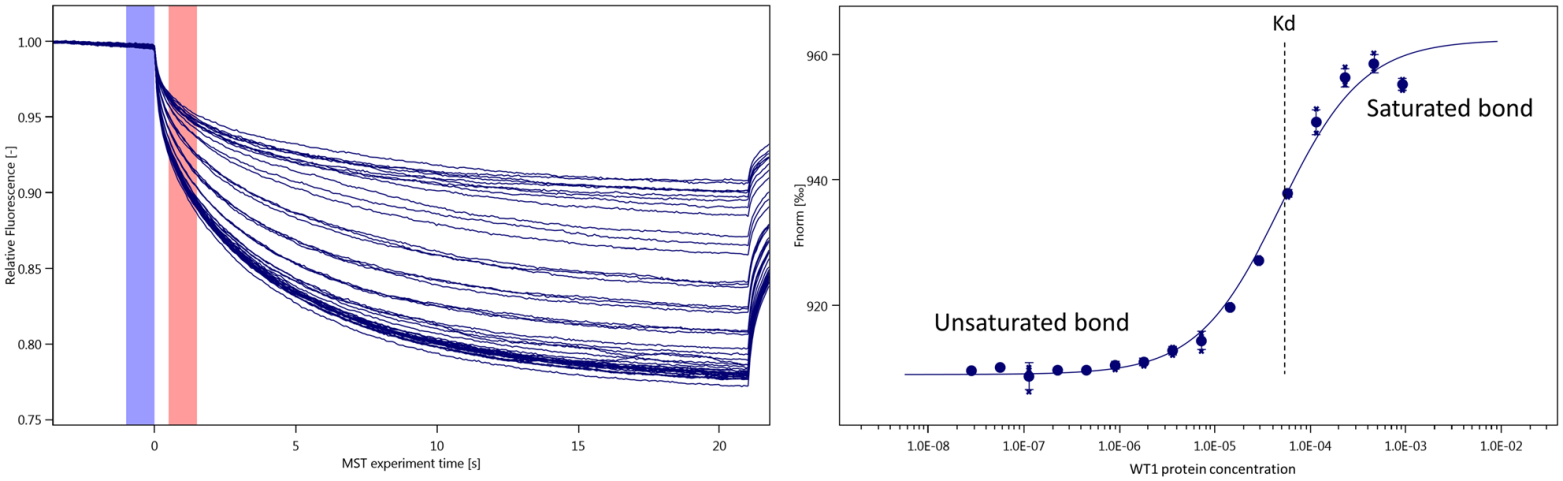
Satisfactory MST traces achieved with optimized MST assay buffer. Left: The curves represent the decrease in fluorescence over time after the application of heat induced by laser. The purple strip marks the baseline while the red strip indicates the point of interest. Right: Normalized fluorescence change in samples of DNA and serially diluted protein. If binding is present, an ”S-affinity curve” is seen and the dissociation constant (dashed line) may be calculated.

Microscale thermophoresis (MST) was performed using a Monolith NT.115 instrument (NanoTemper Technologies) and its built-in software (MO.Control software, NanoTemper Technologies), which includes an intuitive and interactive protocol guidance. The target EGR1 DNA sequence was labelled by cyanine 5 ([Cyanine5]GTGGAGGCG**GCGGGGGCG**GCAGCAACAG produced by Sigma Aldrich) to detect the thermophoresis of the oligo. The assay concentration of the DNA was set up at 80 nM, while the starting (highest) concentration of the protein ranged from 1 to 4 mM. The binding affinity analysis was performed using standard MST capillaries, 20% excitation power (Nano – RED) and medium MST power at room temperature. After optimizing the binding assay buffer (final composition: 50 mM Na_2_HPO_4_.12H_2_O, 150 mM NaCl and 0.005% Tween), satisfactory MST traces were achieved (Fig. 3). The affinity curves for distinct WT1 protein mutants (each measured thrice in a row) were produced by MO.Affinity Analysis software (NanoTemper Technologies).

### Luciferase assay

HEK293 cells were transfected by luciferase reporter gene vector containing *ACTN1* promoter and *WT1* wild type, *WT1* p.Gln447Pro or *WT1* p.His450Arg vectors. All samples were prepared in triplicates and the assay was repeated in six independent measurements. All details of the plasmid production, transfection of HEK293 cells and luciferase assay are available in Supplementary information.

### Statistics

The average dissociation constant (Kd) was calculated from triplicates of each measured WT1 protein and the statistical significance of the difference in mean Kds was then verified by a two-sample t-test computed in R software^47^.

### Ethics approval

The study was approved by the institutional Ethics Committee of Motol University Hospital.The study subjects and their guardians provided their informed consent in writing.

## Supplementary Information

Supplementary Information.

## Data Availability

The datasets generated during and/or analyzed during the current study are available from the corresponding author on reasonable request.
