# Supplementary material for: Alteration in DNA-binding affinity of Wilms tumor 1 protein due to WT1 genetic variants associated with steroid - resistant nephrotic syndrome in children
Source: Sci Rep. 2022 May 24;12:8704. doi: 10.1038/s41598-022-12760-x (PMC9130146; doi:10.1038/s41598-022-12760-x)
Supplement: Supplementary file 1 — Supplementary Information. [file 41598_2022_12760_MOESM1_ESM.pdf]

Supplementary Information

Alteration in DNA-binding affinity of Wilms tumor 1 protein due to *WT1* genetic variants associated with steroid – resistant nephrotic syndrome in children

Martin Bezdicka[1*], Filip Kaufman[2], Ivana Krizova[2], Alzbeta Dostalkova[2], Michaela Rumlova[2], Tomas Seeman[3], Karel Vondrak[3], Filip Fencl[3], Jakub Zieg[3], Ondrej Soucek[1]

[1] Vera Vavrova Lab/VIAL, Department of Pediatrics, Second Faculty of Medicine, Charles University and Motol University Hospital, Prague, Czech Republic

[2] Department of Biotechnology, University of Chemistry and Technology, Prague, Czech Republic

[3] Department of Pediatrics, Second Faculty of Medicine, Charles University and Motol University Hospital, Prague, Czech Republic

***Corresponding author**

Martin Bezdicka, Msc.

Vera Vavrova Lab/VIAL, Department of Pediatrics, Second Faculty of Medicine, Charles University and Motol University Hospital

V Uvalu 84, 150 00

Prague – Czech Republic

Telephone number: +420 257 296 763

Email: Martin.Bezdicka@fnmotol.cz

Preparation of WT1 proteins

Plasmid production

All DNA manipulations were carried out using standard subcloning techniques, and plasmids were propagated in E. coli DH5α. The (–KTS) isoform of the WT1 gene was obtained by reverse transcription of total RNA isolated from A549 cells by RNeasy mini kit (Qiagene). Reverse transcription was performed by RevertAid First Strand cDNA Synthesis Kit (Thermo Scientific), using Oligo (dT)18 primer (Thermo Scientific). The WT1 gene was amplified from the obtained cDNA using 5´forward AAA**GAATTC**ATGGGCTCCGACGTGCGGGACCTG and 3´reverse TTT**AAGCTT**TCAAAGCGCCAGCTGGAGTTTGGTC primers, encoding EcoRI and HindIII restriction sites respectively. An EcoRI-HindIII fragment, encoding the (-KTS) variant of WT1 was then subcloned into pUC19. The selected mutations of WT1 were introduced into a (-KTS)WT1pUC19 vector employing Efficient Mutagenesis Independent of Ligation (EMILI[1, 2]), using the primers given in Supplementary Table S1. Following mutagenesis verification by sequencing, the 999-1270 nts fragments, corresponding to zinc fingers 2-4 of WT1 of either wild type or mutant sequence were amplified using 5´ forward AAAGCTAGCGAGAAACCATACCAG and 3´ reverse TTTCTCGAGTTATCTCTGATGCATG primers, encoding NheI and XhoI restriction sites respectively, and cloned into pET22b. The resulting WT1pET22b vectors were verified by sequencing.

Supplementary Table S1

5´ WT1 ZF2 C11Y	CTTCAAGGACTATGAACGAAGGTTTTCTCGTTC
3´ WT1 ZF2 C11Y	CCTTCGTTCATAGTCCTTGAAGTCACACTGG
5´ WT1 ZF2 R17P	GGTTTTCTCCTTCAGACCAGCTCAAAAGAC
3´ WT1 ZF2 R17P	GGTCTGAAGGAGAAAACCTTCGTTCAC
5´ WT1 ZF2 Q25P	CAAAAGACACCCAAGGAGACATACAGGTGTG
3´ WT1 ZF2 Q25P	ATGTCTCCTTGGGTGTCTTTTGAGCTGGTCTG
5´WT1 ZF3 R10W	AACTTGTCAGTGGAAGTTCTCCCGGTCCGACC
3´ WT1 ZF3 R10W	GGGAGAACTTCCACTGACAAGTTTTACACTGGA
5´ WT1 ZF3 R14W	GAAAGTTCTCCTGGTCCGACCACCTGAAGACC
3´ WT1 ZF3 R14W	GGTCGGACCAGGAGAACTTTCGCTGACAAG
5´ WT1 ZF3 D16N	CTCCCGGTCCAACCACCTGAAGACCCACACC
3´ WT1 ZF3 D16N	CTTCAGGTGGTTGGACCGGGAGAACTTTCGC
5´ WT1 ZF3 H21R	CTGAAGACCCGCACCAGGACTCATACAGGTG
3´ WT1 ZF3 H21R	GAGTCCTGGTGCGGGTCTTCAGGTGGTCGGAC
5´ WT1 ZF3 R10stop	AACTTGTCAGTGAAAGTTCTCCCGGTCCGACC
3´ WT1 ZF3 R10stop	GGGAGAACTTTCACTGACAAGTTTTACACTGGA
5´ WT1 ZF2 H28R	CCAAAGGAGACGTACAGGTGTGAAACCATTCC
3´ WT1 ZF2 H28R	CACACCTGTACGTCTCCTTTGGTGTCTTTTGAG

Protein production

The E. coli BL21 (DE3) CodonPlus RIL strain was transformed by wild type or each mutant WT1 plasmid by using a heat-shock (1 minute at 42 °C). After one hour of incubation in liquid LB Broth medium at 37°C the bacteria were seeded on an agar plate containing 100 µg/ml Ampicillin for selection. The plates were incubated at 37°C overnight, and the next day all grown colonies were moved to liquid LB Broth medium containing 100 µg/ml Ampicillin. The bacteria were incubated for a minimum of 2 hours at 37°C while shaking (220 RPM), until achieving an OD of 0.6 – 0.8. IPTG (the activator of lac operon) was then added to the cell suspension and the incubation with shaking was continued for an additional 4 hours. Cell suspension was centrifuged (10 min, 10 000 x g) and the pellet was frozen at -20°C. The protein expression was verified by the standard SDS-PAGE method using 18% acrylamide gel and 1X Tris-Glycine as a running buffer.

Expression and purification

The isolation of WT1 proteins from the bacterial pellet was achieved by using buffers with increasing concentrations of sodium chloride. All buffers had a pH of 8 and contained 50 mM $Na_2HPO_4.12H_2O$, 0.1% 2-Mercaptoethanol and sodium chloride in an increasing concentration (buffer A 50 mM, buffer B 0.5 M, buffer C 1.0 M and buffer D 2.0 M). The frozen cell pellets were resuspended in buffer A containing 1 mM lysozyme powder, Thermo Halt Protease Inhibitor Cocktail 100X (1000x dilution) and DNAse I (5000x dilution). The suspension was stirred for 20 minutes on ice before 10% sodium deoxycholate was added (0.15% final concentration). The suspension was sonicated (3x 45 seconds; amplitude 40 %; KE76 probe; 3.75 kJ) and centrifuged (15 minutes, 4°C, 30 000 x g). The supernatant was transferred to a clean bottle and left on ice. The previously centrifuged pellet was resuspended in buffer B and the procedure was repeated with buffers C and D respectively. At the end, the pellet was discarded. The presence of the WT1 protein in each supernatant was verified by the standard SDS-PAGE method using 18% acrylamide gel and 1X Tris-Glycine as a running buffer. Supernatants containing WT1 protein were precipitated by 70% saturation of ammonium sulphate on ice. After precipitation the saturated suspensions were centrifuged (30 min, 4°C, 30 000 x g), supernatants were discarded and all pellets were resuspended in buffer A. The resuspended suspension was poured onto the dialysis membrane (Spectra/Por 3 Standard RC Tubing 3.5 kDa). Dialysis was performed overnight in buffer A at 4 °C. The next day the dialyzed sample was centrifuged (30 min, 4°C, 30 000 x g) and the supernatant was preserved on ice (the pellet was discarded).

The prepared sample was subjected to fast liquid protein chromatography (FPLC, ÄKTA pure protein purification system with Unicorn software) to separate the proteins according to their ion charge (HiPrep SP FF 16/10 by Cytiva, strong cation exchange chromatography column). The procedure was performed according to the manufacturer’s instructions, the binding buffer was buffer A and elution was achieved by increasing the gradient of high salt buffer D in the system (all buffers used in the FPLC system were filtrated and degassed). The fractional samples were selected according to UV signal in

Unicorn software and verified by SDS-PAGE gel for WT1 protein presence. The verified fractions were concentrated by centrifugation (Amicon Ultra 15 ml (3000 kDa); 15 min, 4°C, 4000 x g) until the volume was 8 mL. The protein was then separated using high-resolution preparative gel filtration chromatography in a running buffer (50 mM $Na_2HPO_4.12H_2O$, 1 M NaCl, 10 µM $ZnCl_2$, 0.1 % 2-Mercaptoethanol, pH = 8). A HiLoad 26/600 Superdex 200 pg by Cytiva column was prepared according to the manufacturer's instructions. Fractionation was performed with the running buffer in the system and fractions were selected according to UV signal in Unicorn software and verified by SDS-PAGE gel for WT1 protein presence (Supplementary Figure 1). The verified fractions were concentrated by centrifugation (Amicon Ultra 15 ml (3000 kDa); 15 min, 4°C, 4000 x g) until the volume was 6 mL. The concentration of purified WT1 proteins was calculated, and before the MST experiment the samples were concentrated 60 times to approximately 100 µL. The sample buffer was exchanged for the MST binding assay buffer and the final assay concentration was calculated.

Supplementary Figure 1

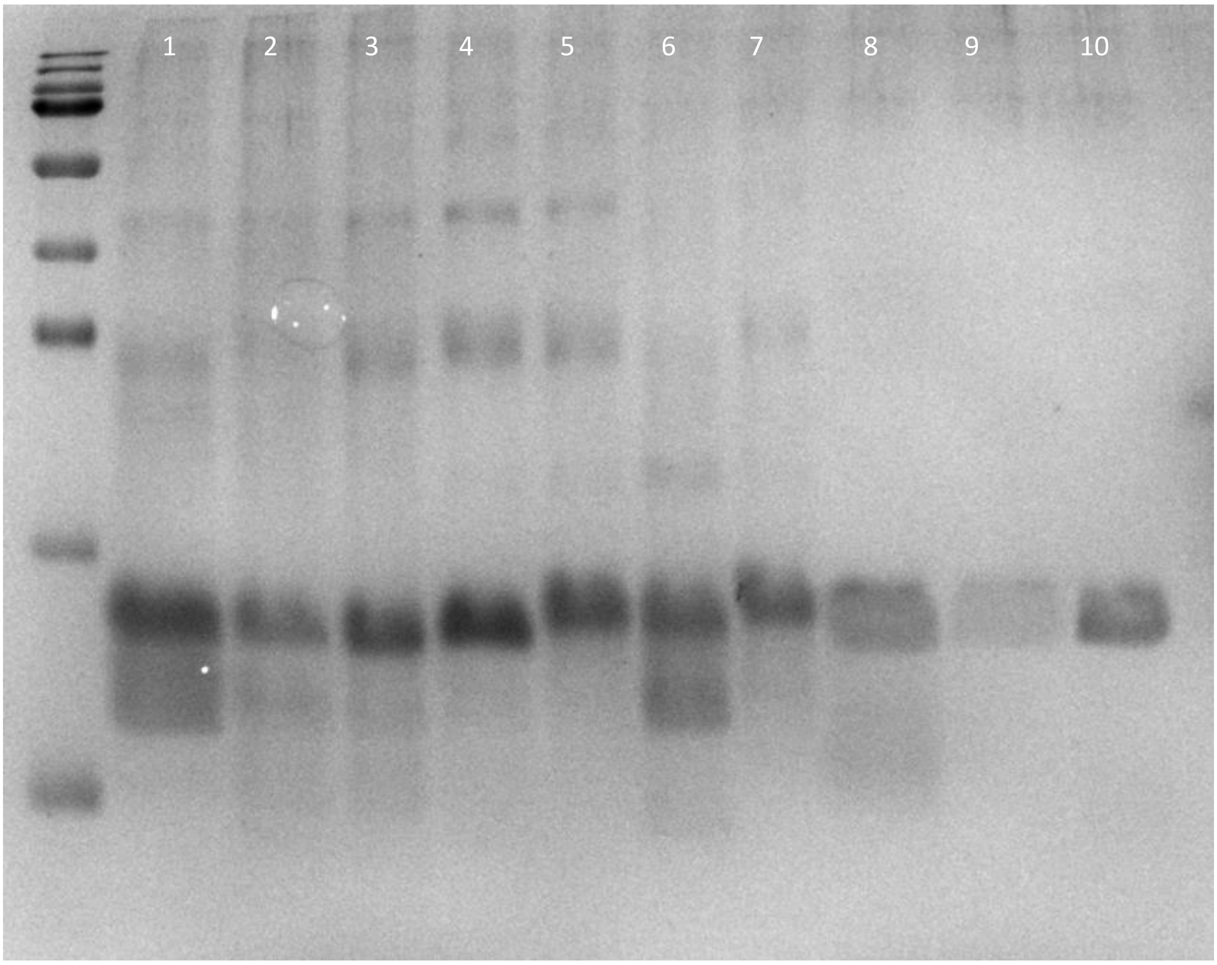

The SDS-PAGE gel with WT1 proteins (zinc-fingers 2-4) loaded in the following order: wild type (1), p.Cys433Tyr (2), p.Arg439Pro (3), p.Gln447Pro (4), p.Asp469Asn (5), p.Arg467Trp (6), p.His474Arg (7), p.His450Arg (8) and p.Arg463Ter (9, 10). The final size of proteins is below 15 kDa (the gel was imaged using Uvitec Alliance 4.7).

Luciferase assay

The luciferase vector pGL4.32 (Promega) was digested by restriction enzymes NheI a HindIII according to commercial protocol (New England Biolabs) and then the promoter of human *ACTN1* gene (NM_001130004) containing *WT1* binding DNA domain (GCGGGGGCG) was inserted into the vector (whole sequence of the insert is in Supplementary Table S2). Two *WT1* variants (p.Gln447Pro, p.His450Arg) were produced in the human *WT1* expression plasmid (RG221271, OriGene) by site-directed mutagenesis using protocol for Platinum™ SuperFi II DNA Polymerase (Thermo Scientific; primers are shown in Supplementary Table S2). The transformation was performed in *DH5α* competent cells (Thermo Scientific). All plasmids were verified by Sanger sequencing. The transfection of HEK293 cells (Sigma-Aldrich) was performed in a 12-well plates (150 000 cells/well) by FuGENE 6 protocol (Promega) with ratio of FuGENE:DNA 3:1. Negative control represented cells transfected with no vectors. The maximum recommended total plasmid concentration was applied (i.e., 2 µg/reaction). The concentrations of individual plasmids were as follows: 0.9 µg of *WT1* plasmid, 1 µg of *ACTN1* pGL4.32 vector and 0.1 µg of *Renilla* luciferase control reporter vector (Promega). All samples were prepared in triplicates (wt, p.Gln447Pro, p.His450Arg and negative control) and repeated in six independent experiments. After two days of incubation, Dual-Luciferase® Reporter Assay System (Promega) protocol was followed. Luminescence was measured by the FLUOstarOmega microplate reader (BMG Labtech) with the program setting as per the Dual-Luciferase® Reporter Assay System protocol recommendation. The presence of WT1 proteins in HEK293 cells was verified by Western Blot (Supplementary Figure 2) using primary turbo GFP antibody (TA150041, OriGene) and secondary polyclonal antibody (A16066, Thermo Scientific).

Supplementary Table S2: Forward and reverse sequences used for plasmid production

ACTN1 promoter sequence	Forward:<br><br>CTAGCCAGCCCAGCCCAGCCCTA**GCGGGGGCG**CACGCCAGGGCAGCAGCCGTTGCTCAGAGA-GAAGGTGGAGGAAGAAATCCAGACCCTAGCACGCGCGCACCATCATGGACCATA
	Reverse:<br><br>AGCTTATGGTCCATGATGGTGCGCGCGTGCTAGGGTCTGGATTTCTTCCTCCAC-CTTCTCTCTGAGCAACGGCTGCTGCCCTGGCGTG**CGCCCCCGC**TAGGGCTGGGCTGGGCTGG
p.Gln447Pro primer	Forward: GCTCAAAAGACACCCAAGGAGACATACA
	Reverse: TGTATGTCTCCTTGGGTGTCTTTTGAGC
p.His450Arg primer	Forward: CACCAAAGGAGACGTACAGGTGTGAAA
	Reverse: TTTCACACCTGTACGTCTCCTTTGGTG

Supplementary Figure 2

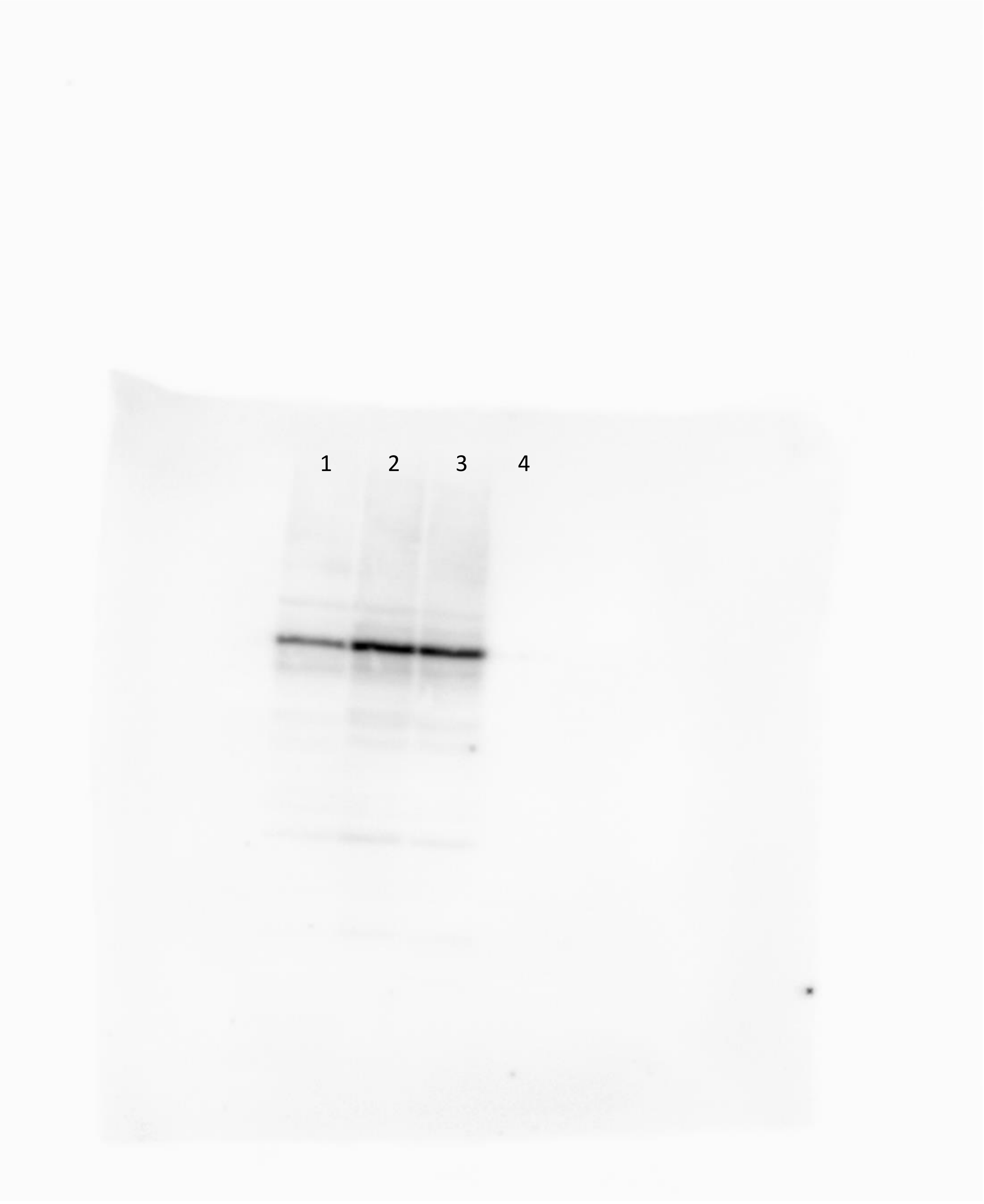

The western blot membrane with WT1 proteins from transfected HEK293 cells used in luciferase assay loaded in the following order from the left: WT1 wild type (1), WT1 p.His450Arg (2), WT1 p.Gln447Pro (3), vector free HEK293 (4). The final size of proteins is 80 – 90 kDa (WT1 56 kDa, turbo GFP 26 kDa; the membrane was imaged using Uvitec Alliance 4.7).

<u>Supplementary references</u>

1. Füzik, T., Ulbrich, P., Ruml, T. Efficient Mutagenesis Independent of Ligation (EMILI). *J Microbiol Methods* **106**, 67-71 (2014). doi:10.1016/j.mimet.2014.08.003
2. Dostálková, A., Kaufman, F., Křížová, I., Vokatá, B., Ruml, T., Rumlová, M. In Vitro Quantification of the Effects of IP6 and Other Small Polyanions on Immature HIV-1 Particle Assembly and Core Stability. *J Virol* **94 (20)**, (2020). doi:10.1128/jvi.00991-20
